# Unveiling Smoking Behavior Amid the COVID-19 Pandemic: A Cross-Sectional Study in the United Arab Emirates

**DOI:** 10.7759/cureus.89040

**Published:** 2025-07-30

**Authors:** Abderrahman Belfakih, Ibraheem Mohammed, Dhuha Zbala, Mohamad Chakaki, Sana Al-Karam, Shatha Al-Sharbatti

**Affiliations:** 1 General Medicine, Gulf Medical University, Ajman, ARE

**Keywords:** changes during covid, chronic smoker, community development, covid-19, covid-19 impact on society, covid-19 in the uae, gender comparison, smoking and covid-19

## Abstract

Objectives

This study aimed to assess the prevalence of smoking among adults residing in the United Arab Emirates (UAE) during the COVID-19 pandemic. Secondary objectives were to identify self-reported changes in smoking behavior, assess associations between smoking and selected epidemiological factors, and determine key predictors for smoking during this period.

Methods

The research team designed and distributed a meticulous questionnaire covering sociodemographic characteristics, lung health, medical and family history, and factors associated with smoking. The instrument was validated by two specialist physicians and a faculty member specializing in behavioral science. The questionnaire gathered information on smoking status, changes in smoking habits during the COVID-19 pandemic, and related factors.

Results

The analysis of 463 participants revealed a smoking prevalence of 28.3% (n = 129/453). During the COVID-19 pandemic, 79.4% (n = 85/107) of smokers who responded to this question reported altering their behavior. Among those who changed their smoking method (n = 11), the primary switch was to vaping. The prevalence of smoking among participants who had contracted COVID-19 was 20% (n = 26/129). Significant age-related differences were observed, with the highest smoking rates found in individuals aged 35 and older at 50.0% (n = 29/58). Prevalence was slightly higher among individuals from non-Eastern Mediterranean regions at 29.1% (n = 39/134) compared to 27.4% (n = 90/329) for those from the Eastern Mediterranean. A high body mass index (BMI) was common among smokers, with 52.5% (n = 31/59) having a BMI over 30. Cigarette smoking was the most common method, preferred by 69.8% (n = 90/129) of smokers. Notably, 74% (n = 94/127) of smokers reported having attempted to quit. The smoking rate among non-healthcare professionals was 54.0% (n = 34/63), significantly higher than the 25.8% (n = 8/31) among healthcare professionals.

Significant associations were found between smoking and age (p < 0.0001), gender (p < 0.0001), occupation (p < 0.0001), living situation (p < 0.0001), family history of smoking (p < 0.0001), chronic disease status (p < 0.0001), and BMI (p < 0.0001). Logistic regression identified gender (adjusted odds ratio (AOR): 3.781, p < 0.0001), being a non-health science student (AOR: 3.717, p < 0.0001), living alone (AOR: 4.948, p < 0.0001), having a family history of smoking (AOR: 3.613, p < 0.0001), and obesity (AOR: 2.692, p = 0.008) as significant predictors of smoking. Smokers also reported a significantly higher prevalence of prior COVID-19 infection compared to non-smokers (20.5% vs 12.0%, p < 0.05).

Conclusion

The smoking prevalence of 28.3% among adults in the UAE during the pandemic represents a significant public health concern. Key predictors for smoking included gender, occupation, living alone, family history, and obesity. The finding that a majority of smokers have attempted to quit presents a crucial "window of opportunity" for implementing effective, behavior-directed strategies to support smoking cessation.

## Introduction

Smoking remains one of the most pressing public health challenges, contributing significantly to global morbidity and mortality [1,2]. Tobacco smoke contains thousands of chemicals, including numerous known carcinogens that contribute to the development of cardiovascular disease, respiratory illnesses, and various cancers [3]. Despite global tobacco control measures, smoking prevalence remains high. In response, the WHO has implemented several strategies, including non-recruitment policies for smokers to reduce workplace exposure [4] and the evidence-based MPOWER policy framework to reinforce public health efforts [5].

The adverse health effects of smoking span multiple systems. It is a significant predictor of atherosclerotic cardiovascular disease, including ischemic heart disease and stroke [6,7]. Smoking has also been linked to gastrointestinal conditions such as Crohn’s disease [8,9]. Additionally, smoking exacerbates insulin resistance and impairs insulin secretion, making it an independent risk factor for type 2 diabetes [10]. The addictive nature of nicotine compounds these effects, reinforcing dependency and hindering cessation efforts [11].

The respiratory system bears a substantial burden from smoking-related damage, including chronic obstructive pulmonary disease (COPD), lung cancer, and reduced lung function [12]. During the COVID-19 pandemic, smoking behaviors shifted significantly in response to heightened psychological stress and social restrictions. Studies from this period reported that many smokers altered their consumption habits, either increasing usage, decreasing intake, or switching to alternative forms such as vaping [13-15].

In the United Arab Emirates (UAE), approximately 900,000 adults are estimated to smoke daily [16]. However, regional studies examining the behavioral patterns and health indicators associated with smoking remain limited [17]. The UAE's unique demographic profile, with a large and diverse expatriate population, makes it a critical case study for understanding behavioral health trends that may be applicable to other multicultural societies and global hubs. While research shows maternal smoking affects fetal lung development [18], few UAE-based studies have provided comprehensive, locally relevant investigations into smoking behaviors [19]. This context, combined with the unique psychosocial stressors of the COVID-19 pandemic [20], highlights a critical gap in the literature.

To address this gap, the primary objective of this study was to determine the prevalence of smoking among adults in the UAE during the COVID-19 pandemic. Secondary objectives were to (1) identify self-reported changes in smoking behavior, (2) assess associations between smoking and sociodemographic factors, and (3) determine key predictors of smoking in this population.

## Materials and methods

A cross-sectional study was conducted using a convenience sample of 463 adults residing in the UAE. Participants aged 18 years and older were recruited online between March 2020 and September 2020 via university email networks and social media community forums. The required sample size was determined using established prevalence rates for tobacco use in the region, as documented in major public health reports and prior local studies [16,19].

A questionnaire was designed by the research team to collect data across several key domains. The development of questions covering sociodemographics, smoking-related factors, and health history was informed by domains used in major public health surveys, such as those detailed in the US Surgeon General's Reports on tobacco use [21]. To ensure content validity, the final instrument was reviewed by two specialist physicians and one faculty member with a specialization in behavioral science. A participant's history of COVID-19 infection was determined via a direct self-report question in the survey. The full questionnaire is available from the corresponding author upon reasonable request.

The study was initiated after receiving approval from the Institutional Review Board (IRB). Informed consent was obtained from all participants prior to their inclusion in the study. An information sheet outlining the study's objectives, benefits, risks, and the right to withdraw was presented, and participants provided consent by proceeding with the anonymous online questionnaire. Data access was restricted to the research team and authorized faculty.

Data were analyzed using IBM SPSS Statistics for Windows, Version 26.0 (IBM Corp., Armonk, NY). Descriptive statistics were used to summarize participant characteristics. The chi-square (χ^2^) test was used to assess associations between smoking status and various categorical factors. Finally, a binary logistic regression analysis was conducted to identify significant predictors of smoking behavior. A p-value of <0.05 was considered statistically significant.

## Results

The study sample consisted of 463 adults residing in the UAE. The overall prevalence of smoking was 27.9% (n = 129/463). Among participants who had previously tested positive for COVID-19 (n = 127), the prevalence of smoking was 20.5% (n = 26). Smoking was most prevalent among individuals aged 35 years and above, where 50% (n = 29) of that age group were identified as smokers. A chi-square test revealed a significant association between age and smoking status, χ^2^(2, N = 463) = 9.38, p = 0.009, Cramér’s V = 0.14.

Regional differences were also observed: 29.1% (n = 39) of non-Eastern Mediterranean participants were smokers, compared to 27.4% (n = 90) of Eastern Mediterranean participants. Gender-based analysis showed significantly higher smoking rates in males (48.5%, n = 98) compared to females (11.9%, n = 31).

A significant association was found between BMI group and smoking status (χ^2^(3, N = 453) = 27.86, p < 0.001, Cramér’s V = 0.25). Smoking prevalence was highest among participants with a BMI over 30, at 52.5% (n = 31). Cigarettes remained the preferred smoking method for 69.8% (n = 90) of smokers, and 74% (n = 94) reported having made at least one attempt to quit in their lifetime. Furthermore, a significant association was found between smoking status and a history of COVID-19 infection (p < 0.05). The prevalence of a prior positive test was notably higher among smokers (20.5%) compared to non-smokers (12.0%).

Professional background showed a marked difference: 54% (n = 34) of non-healthcare professionals were smokers compared to 25.8% (n = 8) of healthcare professionals. Smoking status was significantly associated with occupation (χ^2^(2, N = 463) = 14.05, p < 0.001, Cramér’s V = 0.17).

A significant association was found between the perceived risk of chronic complications and smoking status (χ^2^(2, N = 463) = 7.79, p = 0.02, Cramér’s V = 0.13). Similarly, perception of COVID-19 risk showed a weaker but significant association with smoking (χ^2^(2, N = 463) = 7.13, p = 0.028, Cramér’s V = 0.12).

Overall, these findings highlight critical demographic and behavioral factors associated with smoking, offering key targets for intervention and future research, as summarized in Table 1.

**Table 1:** Comparison of Demographic and Health Characteristics Between Smokers and Non-smokers

Characteristic	Smokers (n = 129)	Non-smokers (n = 334)	p-value
Age Group			
20-24	76 (58.9%)	273 (81.7%)	<0.0001
25-29	14 (10.9%)	15 (4.5%)	-
30-34	10 (7.8%)	17 (5.1%)	-
>35	29 (22.5%)	29 (8.7%)	-
Gender			
Male	98 (76.0%)	104 (31.1%)	<0.0001
Female	31 (24.0%)	230 (68.9%)	-
History of Chronic Disease			
Yes	31 (24.0%)	26 (7.8%)	<0.0001
No	98 (76.0%)	308 (92.2%)	-
BMI Category			
<18.5 (Underweight)	6 (4.7%)	21 (6.3%)	<0.0001
18.5-24.9 (Normal)	46 (35.7%)	187 (56.0%)	-
25-29.9 (Overweight)	45 (34.9%)	89 (26.6%)	-
>30 (Obese)	31 (24.0%)	28 (8.4%)	-
History of COVID-19			
Yes	26 (20.5%)	40 (12.0%)	<0.05
No	101 (79%)	-	-

## Discussion

The smoking prevalence in this study was 27.9%. This rate is notably higher than the global average but is consistent with the high prevalence of tobacco use documented across the WHO Eastern Mediterranean Region [1,22]. Most participants in our study (64.3%) began smoking between the ages of 15 and 19. This finding strongly aligns with extensive evidence identifying adolescence and young adulthood as the critical period for smoking initiation, a pattern detailed in major public health reports like the US Surgeon General's Report on youth and tobacco use [21]. This pattern is particularly concerning, as early initiation is often linked with higher nicotine dependence and lower rates of successful cessation later in life [23].

The COVID-19 pandemic appeared to be a pivotal event for smokers, with a substantial portion (79.4%) reporting changes in their smoking behavior. Notably, the most common change was a decrease in smoking (36.4%). This contrasts with findings from a UK study where only 20% of smokers reported a decrease during the same period [14]. Conversely, 24.3% of smokers in our sample reported an increase in smoking. This may be linked to the psychological distress of the pandemic, a finding that aligns with research connecting poor mental health and depressive symptoms with increased tobacco use [20,24]. Furthermore, 13.2% of those who changed their habits reported switching their smoking method, with many transitioning to vaping. This trend is consistent with findings from Italy, where an increase in e-cigarette use was observed during pandemic lockdowns [13].

Among the smokers in our sample, 20.5% had a history of COVID-19 infection. For sociodemographic factors, age was strongly associated with smoking (p < 0.0001), with the highest prevalence (50.0%) observed among individuals aged 35 and older. This trend of smoking rates peaking in middle-adulthood is similar to patterns observed in both the United States and Korea [6,25]. The finding that smokers in our sample were more likely to report a history of COVID-19 infection reinforces the public health understanding of smoking as a risk factor for susceptibility to respiratory illnesses.

Gender differences were also highly significant (p < 0.0001), with a prevalence of 48.5% among males compared to just 11.9% among females. This reflects the well-documented global gender gap in smoking, where men have historically had substantially higher rates of tobacco use than women, a pattern confirmed in numerous global reports [26,27].

Limitations

This study has several limitations. First, the use of a convenience sampling method and an online questionnaire may introduce selection bias, limiting the generalizability of our findings to the broader UAE population. Second, the reliance on self-reported data is subject to recall and social desirability biases. Third, the cross-sectional design precludes the establishment of causal relationships; therefore, all findings must be interpreted as associations, not causal links. This is particularly relevant to the predictors identified in the regression analysis, which represent statistical associations rather than proven causal factors. Finally, the study did not include biochemical verification of smoking status, and while the questionnaire underwent an expert content review, it was not formally pilot-tested for psychometric validation.

## Conclusions

This study provides valuable insights into the dynamics of smoking in the UAE during the COVID-19 pandemic, confirming a significant smoking prevalence and highlighting strong associations with demographic factors such as age, gender, and professional background. A marked disparity in smoking rates between non-healthcare and healthcare professionals underscores a critical need for targeted workplace interventions. While traditional cigarettes remain the preferred method of consumption, the observed shift towards vaping warrants regulatory attention. Crucially, this study revealed a significant desire for cessation, with a substantial majority of smokers having previously attempted to quit. This finding represents a vital "window of opportunity" for public health initiatives to support and motivate individuals on their journey to becoming smoke-free.
